# Postoperative Spontaneous Gastric Perforation in a Ruptured Exomphalos: A Case Report 

**Published:** 2016-04-10

**Authors:** Hassan RAA, Ridzuan AS, Guan TP, Sayed A

**Affiliations:** Department of Pediatric Surgery, Hospital Universiti Sains Malaysia

**Keywords:** Perforation, Gastric, Neonate, Omphalocele

## Abstract

Spontaneous gastric perforation is a rare entity in neonates. We report a case of spontaneous gastric perforation in a neonate operated for in-utero rupture of omphalocele.

## CASE REPORT

At 35th weeks of gestational age (mother 19-year-old, primigravida), a baby girl was delivered via emergency C/S for intrauterine growth retardation and suspicion of strangulation of eviscerated bowel through anterior abdominal wall defect. Her birth weight was 1900 gms and Apgar score was 9 at 1st minute. On examination she had a ruptured exomphalos sac with parts of stomach, small and large intestines lying outside the abdomen (Fig. 1). Protruded bowel was dusky but improved after resuscitation. There was no thickening or matting of bowel. At operation, there was rupture of exomphalos sac measuring 3x3 cm in diameter. Bowel was reduced easily after milking out meconium through anus and primary repair done with umbilicoplasty. She was kept electively ventilated. On postoperative D-2 baby developed abdominal distension and deterioration of her general condition. An emergent abdominal x-ray revealed pneumoperitoneum. Emergency laparotomy done through supra-umbilical transverse incision showed a stomach perforation of 1x1 cm size at the fundus (Fig. 2). Primary repair of perforation was done after ruling out distal obstruction. Baby was extubated from ventilatory support after 2 days and had a speedy recovery. She was discharged in good clinical condition. 

**Figure:**
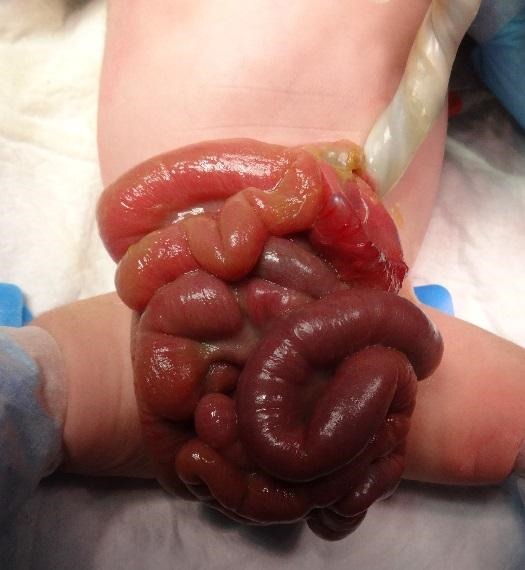
Figure 1: Ruptured exomphalos

**Figure:**
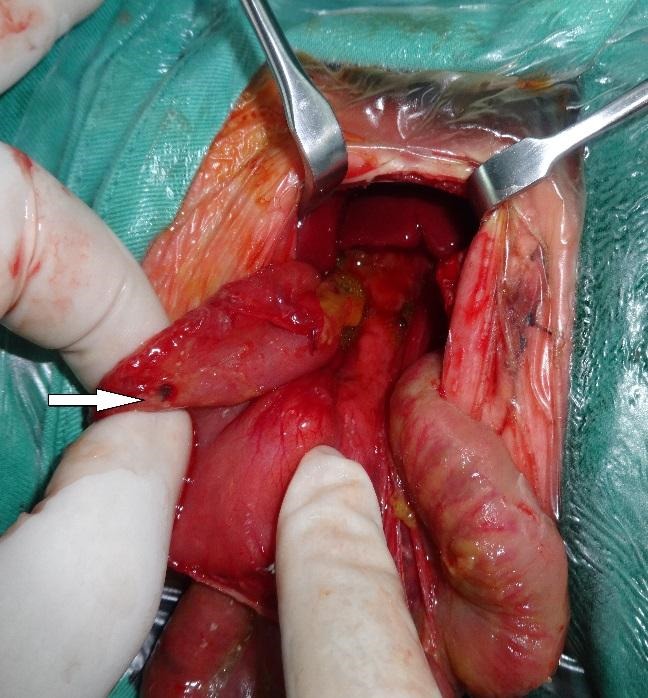
Figure 2: Gastric perforation (arrow)

## DISCUSSION

Three mechanisms have been proposed for gastric perforation (GP) in neonates: traumatic, ischemic, and spontaneous.[1-4] Spontaneous GP is considered secondary to congenital weakness of gastric wall smooth muscles and have been reported in otherwise healthy neonate in between 2nd and 7th day of life [1]. Perforation generally occurs on the fundus and greater curvature of the stomach but may involve the posterior wall as well [2, 3]. Our case had intrauterine rupture of exomphalos sac diagnosed at 35th week of gestation with growth retardation. The fetus was delivered via emergency C-section. The baby had a repair of exomphalos without much delay. She had perforation of stomach on postoperative D-2 when she was on mechanical ventilation. There was no evidence of traumatic injury by orogastric tube or inadvertent injury from vigorous resuscitation. We believe the perforation in the index case is of spontaneous nature as there is no evidence of ischemia or trauma by NG tube or vigorous resuscitation. Few cases of GP due to distal obstruction are reported [5] but not found in the index case.

## Footnotes

**Source of Support:** Nil

**Conflict of Interest:** Nil
